# The Critical Role of Nanoparticle Geometry in Turnover Frequency Calculation

**DOI:** 10.1021/acsmeasuresciau.5c00130

**Published:** 2025-10-14

**Authors:** Zohreh Akbari, Loris Lombardo, Andreas Züttel

**Affiliations:** † Laboratory of Materials for Renewable Energy (LMER), Institute of Chemical Sciences and Engineering (ISIC), Basic Science Faculty (SB), École Polytechnique Fedérale de Lausanne (EPFL) Valais/Wallis, Energypolis, Rue de l’Industrie 17, CH-1951 Sion, Switzerland; ‡ Empa Materials Science & Technology, CH-8600 Dubendorf, Switzerland

**Keywords:** turnover frequency, dispersion, heterogeneous catalysis, supported metal catalysts, catalytic hydrogen combustion

## Abstract

Accurate evaluation and comparison of site-normalized catalytic activity (turnover frequency, TOF) in heterogeneous catalysis require consideration of catalyst nanoparticle (NP) size and geometry. In this study, we systematically quantify the impact of NP geometry on the fraction of surface atoms across FCC, BCC, and HCP crystal structures with various geometries and evaluate the absolute and relative errors introduced by assuming spherical NPs. Using catalytic H_2_ combustion (CHC) over an octahedron Ni catalyst supported on γAl_2_O_3_ as a model experiment, we demonstrate that assuming spherical single-crystal Ni NPs underestimates the fraction of the surface atoms and overestimates TOF by 86%. This discrepancy arises from the miscalculation of surface site availability in spherical approximations. These findings emphasize the need for geometry-specific models to ensure reliable TOF calculations and accurate catalyst performance comparisons in heterogeneous catalysis. We work provide a framework for geometry-dependent TOF calculations, offering new insights into morphology-controlled catalyst design and facet-specific reactivity optimization.

## Graphical abstract

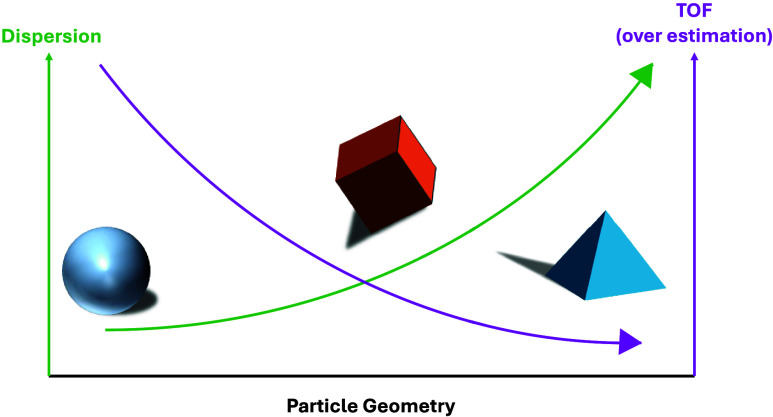

## 1 Introduction

Assessing catalytic performance in heterogeneous catalysis begins with measuring reaction rates.^1^ In most of the heterogeneous catalysis processes, supported metals are used to increase the number of surface sites by utilizing nanoparticles (NPs) on a high surface area support.^2–4^ However, comparing catalytic activity across different catalysts requires identical conditions, particularly in support material properties and NP shape and size. The key question is how to reliably compare the catalytic activity of different supported catalysts?

To address these issues, about 60 years ago, Boudart et al.^5,6^ introduced turnover frequency (TOF) as an effective metric, defined by the number of surface sites and their turnover rate.^7^ However, TOF calculation (eq 1) depends on determining the surface site fraction, known as dispersion (eq 2), which remains challenging due to measurement uncertainties.

1 $$TOF=reaction\;rate\;(\frac{{mol}_{H2}}{{mol}_{catalyst}.s})\times \frac{1}{dispersion}$$

2 $$F\left(\%\right)=\mathrm{fraction}\;\mathrm{of}\;\mathrm{surface}\;\mathrm{atoms}=\mathrm{dispersion}=100\times \frac{N_{S}}{N_{T}}$$

where *F* is the fraction of surface atoms or dispersion and *N*_*T*_ is the total number of atoms in a particle, equal to the sum of the number of bulk (*N*_*B*_) and surface atoms (*N*_*s*_).

Probe gas chemisorption using CO,^8^ O_2_,^9^ N_2_O,^10,11^ and H_2_ ^12^ for metal catalysts and methanol (CH_3_OH) for oxide catalysts,^13^ is a common approach for surface site quantification. These methods quantify the adsorbed probe gas and estimate the number of surface sites based on stoichiometry. However, this approach has several limitations: it assumes the full reduction of metal oxides, which may not be achievable; it relies on a defined NP geometry, often assumed to be spherical;^14^ and it is highly sensitive to experimental conditions, particularly temperature.^15^ When FTIR spectroscopy is used, the accuracy of surface site determination further depends on the molar extinction coefficient (ε) for CO adsorption.^16–18^ Beyond these theoretical and experimental uncertainties, chemisorption methods do not guarantee that all detected surface sites contribute equally to catalysis. Strong reactant adsorption and phase transitions during reactions may render some sites inactive.^6^ Additionally, support materials inevitably adsorb probe gases, introducing further errors. These factors lead to potential over- or underestimation of surface site numbers, impacting TOF accuracy.^1^

An alternative approach is to estimate surface sites from N_2_ adsorption/desorption measurements. Mutschler et al.^19^ combined electron microscopy for NP size determination with BET-derived surface areas to estimate the number of surface atoms. However, this method is more applicable to unsupported catalysts. In supported catalysts, metal NPs can obscure the support surface area and block micropores, making the direct summation of NP and support surface areas inaccurate. Consequently, TOF calculations using this method require excessive approximations.

Given these challenges, a more reliable approach should determine the fraction of surface sites based on NP geometry and size, independent of the support material. This is crucial since NP shape influences not only reaction rates but also cohesive energy,^20,21^ metal–support interaction,^22^ lattice parameters,^23^ and, more importantly, as demonstrated by Zuttel et al.,^24^ H_2_ adsorption and desorption.

Qi et al.^25^ attempted to address this issue by introducing a shape factor, defined as the ratio of the surface area of a nonspherical NP to that of a sphere with the same volume. In their model, a perfect sphere has a shape factor of 1, while other geometries have values greater than 1. They estimated shape factors for tetrahedrons, hexahedrons, and octahedrons, but their method only roughly describes the difference between spheres and polyhedrons. To calculate surface atom numbers, they applied this shape factor to a spherical NP model, which ultimately underestimated surface sites by considering only the largest cross-sectional area per face.

More recently, Nanda et al.^26^ compared four different approaches for estimating surface atoms, but their analysis was limited to spherical NPs.

While these studies provided valuable insights, they highlight the need for a more comprehensive geometric model that accounts for real NP geometries to accurately calculate the TOF.

To address this gap, our research establishes a direct correlation between NP geometry and the fraction of surface atoms, using electron microscopy data, to ultimately reliably calculate the TOF with respect to the geometry of the supported catalyst NPs. We calculate surface, bulk, and total atom counts for Ni NPs with three different crystal structures, assuming an atomic radius of 0.124 nm. The fraction of surface atoms is determined using eq 2 and compared to spherical NP assumptions.

To validate this approach, we conduct catalytic H_2_ combustion (CHC) over Ni-γAl_2_O_3_ catalyst, as a model catalytic experiment. The goal is to refine TOF calculations based on NP geometry, enabling more accurate activity comparisons of different catalysts. This study provides a foundation for geometry-dependent TOF determination, offering a more precise framework for evaluating catalytic performance in heterogeneous catalysis.

While multiple factors contribute to uncertainty in TOF determination, including variability in reaction conditions, NP size and shape distributions, and the dynamic nature of surface sites under reaction conditions, this study addresses a distinct and quantifiable source of a systematic error: the geometric assumptions used to estimate dispersion from NP morphology. The proposed model isolates this contribution and provides a general framework for correcting geometry-induced bias in TOF calculations when morphological data are available.

## 2 Experimental Details

### 2.1 Catalyst Synthesis and Characterization

The 10 wt % Ni on γAl_2_O_3_ support is synthesized by the incipient impregnation method, as described in our previous work.^27^ Aberration-corrected high-angle annular dark-field scanning transmission electron microscopy (AC-HAADF-STEM) is used to measure the size and distribution of the Ni NPs, employing a Thermo Fisher Scientific Spectra200 system at 200 kV. Elemental maps are obtained through energy dispersive spectroscopy (EDX) using Super-X detectors.

The phase transformation and Ni NPs crystallinity of the synthesized catalyst during the reduction procedure are investigated with an in situ X-ray diffraction (Bruker, D8 Discover) setup. In this experiment, a capillary is filled with the catalyst powder and after three cycles of evacuation and filling with H_2_, the capillary is heated up to 400 °C.

### 2.2 Experimental Setup and CHC Catalytic Activity Measurement

The experimental setup has been described in our previous publications.^27,28^ In brief, 200 mg of Ni-γAl_2_O_3_ catalyst with a pellet size of 250–500 μm is fixed by quartz wool in the middle of a plug-flow reactor. After activation at 400 °C under Formier gas (5 vol % H_2_ in N_2_) for 1 h, a mixture of 4 vol % H_2_, 2 vol % O_2_, and the rest N_2_ with a total flow of 20 mL.min^–1^ is fed into the reactor. The temperature ramped up to 360 °C with a heating rate of 1 °C.min^–1^, and the exhaust gas was analyzed by a quadrupole mass spectrometer (QMS). The H_2_ conversion rate (*r*) is determined from H_2_ conversion (*X*_H_2__).

## 3 Methodology

### 3.1 Geometrical Surface Site Calculation

The fraction of surface atoms in a NP is strongly influenced by its crystal structure and geometry. Since catalytic activity occurs at the surface of the catalyst, a precise determination of the fraction of surface atoms is essential for accurate turnover frequency (TOF) calculations. In this study, we employ Van Hardeveld’s approach^29^ to determine *N*_*S*_, *N*_*B*,_ and *N*_*T*_ by relating them to the number of surface atoms along an equivalent edge (τ), which follows a second-order polynomial relationship with *N*_*S*_ (Table 1).

**Table 1:** Formula to Calculate *N_T_*, *N_B_*, and *N_S_* for Different Shapes of Each Crystal Structure^29^

crystal structure	geometry	*N_S_*	*N_B_*	*N_T_*
FCC	tetrahedron	2τ^2^ – 4τ + 4	$\frac{1}{6}\left(\tau^{3}-9\tau^{2}+26\tau -24\right)$	$\frac{1}{6}\left(\tau^{3}+3\tau^{2}+2\tau \right)$
	cube	12τ^2^ – 24τ + 14	4τ^3^–18τ^2^ + 27τ – 14	4τ^3^ – 6τ^2^ + 3τ
	octahedron	4τ^2^ – 8τ + 6	$\frac{1}{3}\left(2\tau^{3}-12\tau^{2}+25\tau -18\right)$	$\frac{1}{3}\left(2\tau^{3}+\tau \right)$
	rhombic dodecahedron	96τ^2^ – 216τ + 122	64τ^3^ – 264τ^2^ + 364τ – 165	64τ^3^ – 168τ^2^ + 148τ – 43
	cubo octahedron	30τ^2^ – 60τ + 32	16τ^3^ – 63τ^2^ + 84τ – 38	16τ^3^ – 33τ^2^ + 24τ – 6
BCC	cube	6τ^2^ – 12τ + 8	2τ^3^ – 9τ^2^ + 15τ – 9	2τ^3^ – 3τ^2^ + 3τ – 1
	octahedron	8τ^2^ – 12τ + 6	$\frac{1}{3}\left(8\tau^{3}-30\tau^{2}+40\tau -21\right)$	$\frac{1}{3}\left(8\tau^{3}-6\tau^{2}+4\tau -3\right)$
	rhombic dodecahedron	12τ^2^ – 24τ + 1 4	4τ^3^ – 18τ^2^ + 28τ – 15	4τ^3^ – 6τ^2^ + 4τ – 1
HCP	hexagonal bipyramid	3τ^2^ – 6τ + 5	$\frac{1}{2}\left(\tau^{3}-6\tau^{2}+13\tau -10\right)$	$\frac{1}{2}\left(\tau^{3}+\tau \right)$
	truncated hexagonal bipyramid	$\frac{1}{2}\left(21\tau^{2}-42\tau +25\right)$	$\frac{1}{4}\left(14\tau^{3}-63\tau^{2}+98\tau -53\right)$	$\frac{1}{4}\left(14\tau^{3}-21\tau^{2}+14\tau -3\right)$

Since different NP geometries exhibit distinct atomic arrangements, the total fraction of surface atoms varies across crystal structures and morphologies. Consequently, a geometry-dependent framework for estimating *F* is necessary to account for these structural effects.

In this regard, we assume each NP is a single particle without agglomeration. The average NP’s size (*D*, nm) is measured from STEM-HAADF images and equated to the maximum length (*L*_max_), which represents the diameter of the circumscribed sphere of each NP.

Given the atomic radius (*r*, nm) of the metal, the vertex length (*a*, nm) is derived from *L*_max_. For certain geometries, the interatomic distance at the vertex (*d*, nm) is also considered for τ calculation. This ensures that atomic spacing and coordination effects are incorporated into the model, capturing deviations from idealized spherical approximations.

Table 2 summarizes the relationships between *L*_max_, *d*, and τ for various geometries, providing a framework for precise atomic quantification. By substituting the τ value into the corresponding polynomial expressions (Table 1), *N*_*S*_, *N*_*B*_ and *N*_*T*_ and subsequently the fraction of surface atoms in each crystal structure and geometry can be determined. This method allows for an explicit comparison of surface site availability across different catalysts.

**Table 2:**
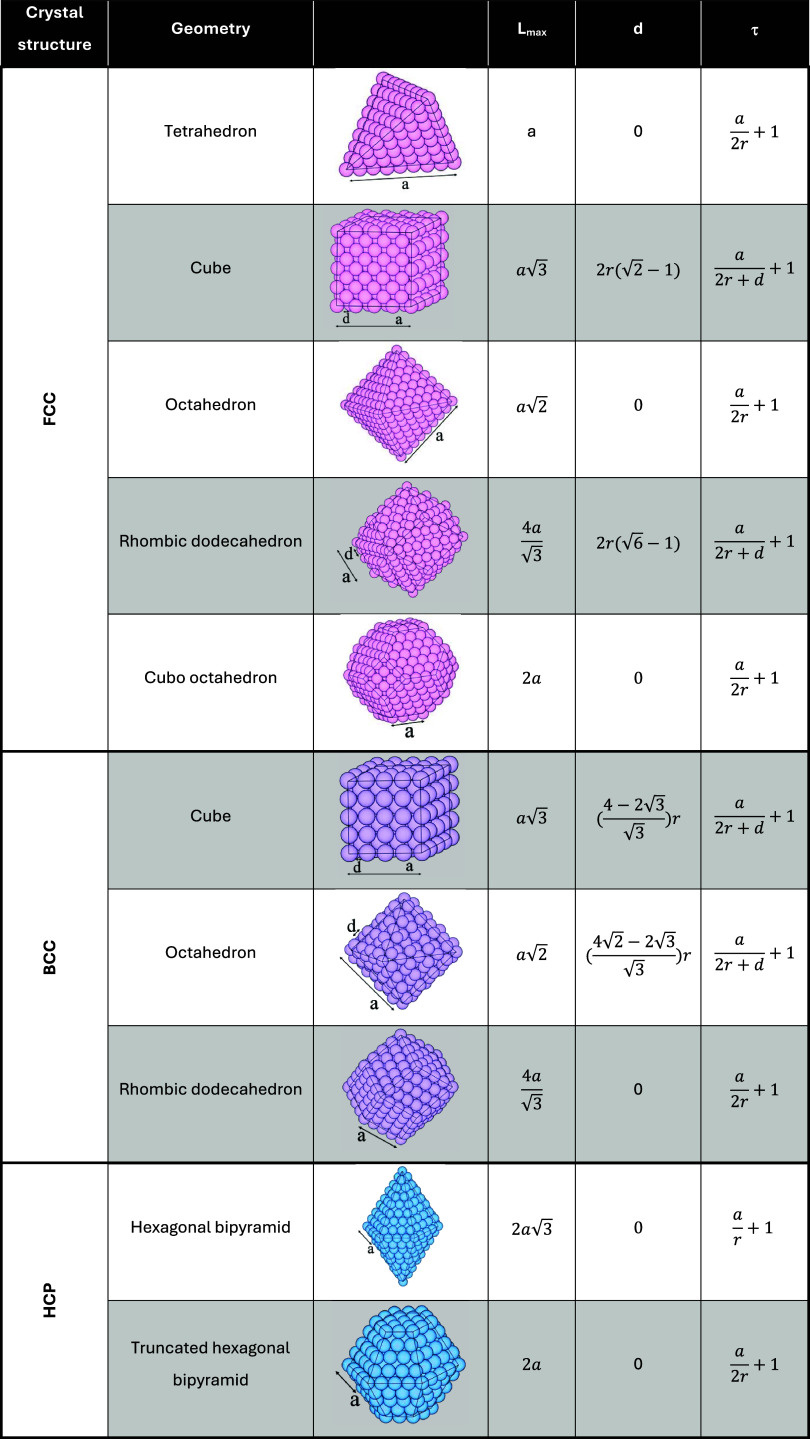
Relationship between *L*_max_, *d*, and τ for Different Geometries of Each Crystal Structure

## 4 Results and Discussion

### 4.1 Accuracy of Surface Atom Fraction Models for Spherical NPs

Three established models, the Qi, Mattson, and Kanaras models, are evaluated for spherical NPs to identify the most accurate approach. Here, the term “accuracy” refers to how well the physical assumptions of each model represent the actual distribution of atoms on the NP surface. This accuracy is relative to these assumptions, and real NPs may deviate from ideal shapes.

#### 4.1.1 Qi Model^25^

In this model, the N*_T_* is determined as the ratio of an NP’s volume to the volume of a single atom. The *N*_*S*_ is then calculated by considering the contribution of the largest cross-sectional area of each atom (π*r* ^2^) to the surface of the NP, leading to the expression in eq 3

3 $$F=100\times \frac{N_{S}}{N_{T}}=100\times \frac{{\left(\frac{D}{r}\right)}^{2}}{{\left(\frac{D}{2\times r}\right)}^{3}}=\frac{8\times r}{D}$$

#### 4.1.2 Mattson Model^30^

Unlike the Qi model, Mattson’s model incorporates mass to determine *N*_*T*_ and *N*_*S*_. Here, *N*_*T*_ is calculated as $\frac{\rho \times \pi }{6\times m}\times D^{3}$ , where *m* is the mass of a single atom and ρ is the density of the metal. The surface of the sphere-shaped NP is assumed to be entirely covered by atoms with no interatomic spacing. Therefore, *N*_*S*_ is calculated by subtracting the number of atoms just below the surface layer ( $\frac{\rho \times \pi }{6\times m}\times {\left(D-4\times r\right)}^{3}$ ) from *N*_*T*_. Consequently, *F* can be calculated by eq 4

4 $$F=100\times \frac{N_{S}}{N_{T}}=100\times \frac{D^{3}-{\left(D-4\times r\right)}^{3}}{D^{3}}$$

#### 4.1.3 Kanaras Model^31^

Kanaras introduced a refinement by incorporating interatomic spacing, where the surface atoms reside within a shell of thickness equal to one unit cell. This shell, with a thickness of one unit cell, has a volume (*V*_shell_) obtained by subtracting the core volume ( $V_{\mathrm{core}}=\frac{\pi }{6}\times {\left(D-l\right)}^{3}$ ) from the total volume of an NP ( $V_{\mathrm{NP}}=\frac{\pi }{6}\times D^{3}$ ). Using the ratio between *V*_shell_ and *V*_NP_, *N*_*S*_ is determined, yielding eq 5

5 $$F=100\times \frac{N_{S}}{N_{T}}=100\times \frac{D^{3}-{\left(D-l\right)}^{3}}{D^{3}}$$

However, even the Kanaras model fails to account for the surface packing density, and as a result, the crystal structure remains unconsidered.

### 4.2 Model Comparison and Selection

Figure 1 illustrates the results of these models for Ni NPs (*r* = 0.124 nm) across a size range up to 20 nm. The Mattson model overestimates *F* due to the assumption of a densely packed atomic layer without interstitial spaces. As the NP size increases above 5 nm, the Qi and Kanaras models approach similar values. However, in smaller NPs, the Qi model introduces an error because it only considers the largest surface fraction of atoms to calculate the *N_S_*. Prior work by Nanda et al.^26^ has also validated the Kanaras model as the most accurate for spherical Au NPs. Consequently, in the rest of this paper, our proposed approach to estimating the *F* will be compared to the Kanaras model. This comparison provides a baseline for evaluating our geometry-dependent corrections, recognizing that the Kanaras model still assumes perfect sphericity.

**Figure 1:**
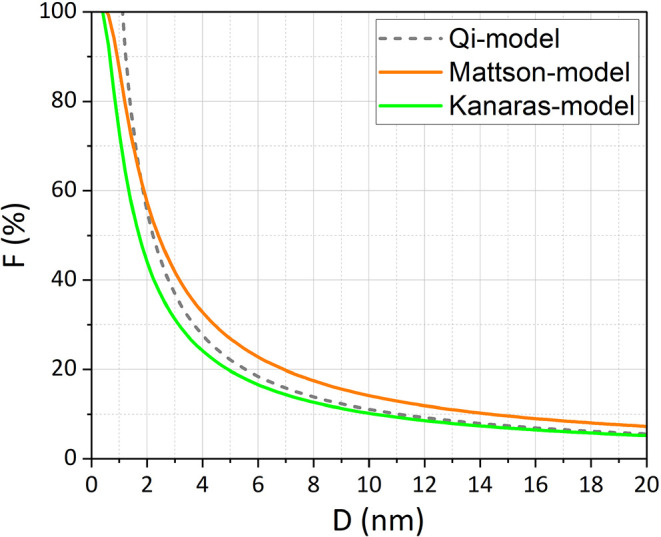
Comparison of the calculated fraction of surface atoms obtained from three different models with *r* = 0.124 nm.

It is essential to note that these models are applicable only to ideal spherical NPs. Spheres have the lowest N_S_ and, consequently, the lowest *F*. Any deviation from spherical symmetry increases *F*, introducing potential errors in TOF calculations. Therefore, a model incorporating real NP geometries is necessary.

### 4.3 Effect of Crystal Structure and Geometry on *F*

#### 4.3.1 FCC NPs

Using the methodology outlined earlier, *F* is calculated for FCC metals across five different geometries, as shown in Figure 2a. As is evident, highly faceted geometries such as tetrahedrons exhibit a significantly larger fraction of surface atoms than compact structures like cuboctahedrons. To quantitatively interpret these differences, we introduce sphericity (Ψ), a parameter (eq 6) that quantifies how closely a three-dimensional shape resembles a perfect sphere (Ψ = 1 for a sphere).

6 $$\Psi =\frac{\pi^{1/3}\times {\left(6\times V\right)}^{2/3}}{A}$$

In this equation, *V* and *A* represent the volume and surface area of the corresponding geometry, respectively.

**Figure 2:**
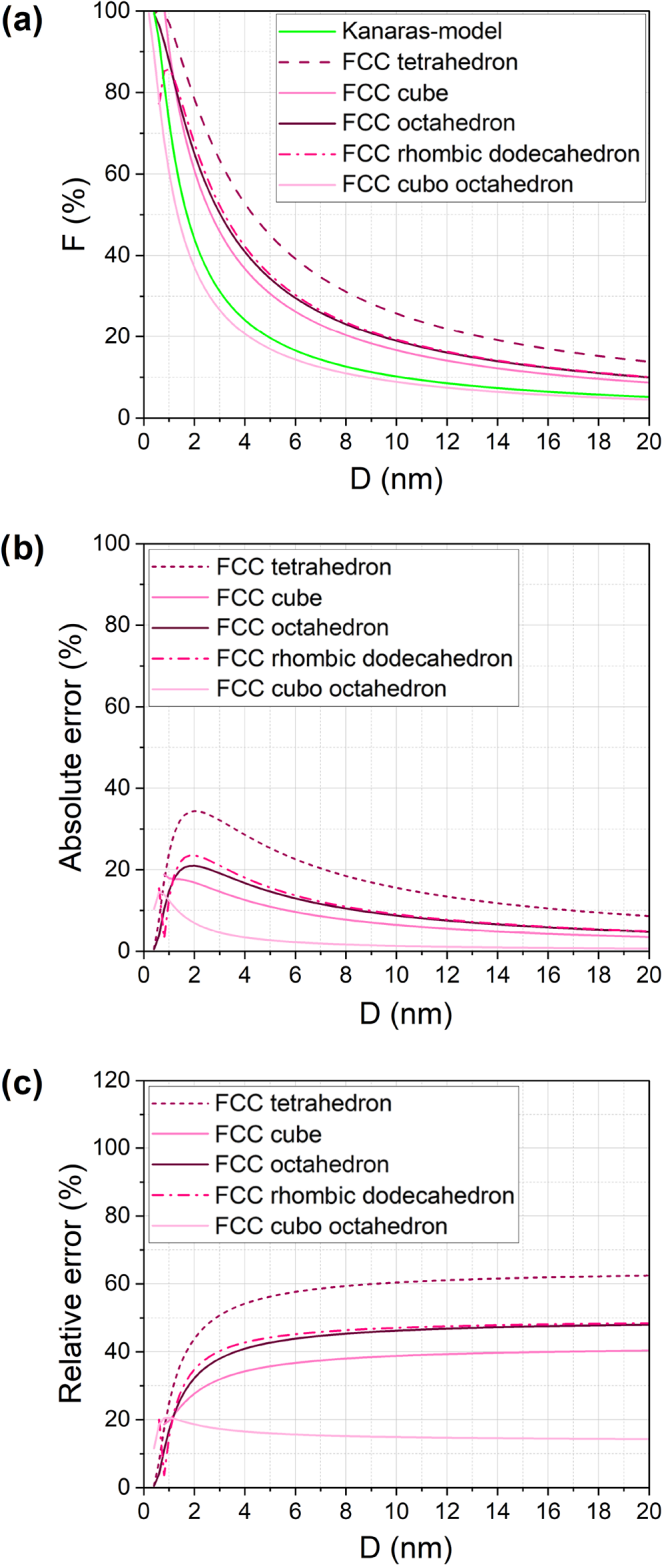
Comparison of (a) calculated fraction of surface atoms for FCC crystal structure with different shapes, (b) absolute error, and (c) the relative error in comparison to Kanara’s model (*r* = 0.124 nm).

Sphericity values for the tetrahedron and cuboctahedron are 0.671 and 0.9, respectively. As the Ψ value approaches 1, *F* predicted by our proposed model converges to the Kanaras model. However, as the NPs shape deviates toward polyhedral structures, *F* increases significantly.

Figure 2b shows the absolute error (eq 7) between our model and the Kanaras model, which reaches a maximum for 2 nm NPs. The relative error (eq 8) increases until leveling off for particles larger than 10 nm, regardless of the NPs’ geometry (Figure 2c).

For tetrahedral FCC NPs, the relative error reaches 60%, while even for the relatively spherical cuboctahedron, the discrepancy is as high as 15%. These findings highlight the necessity of incorporating geometry-dependent corrections in TOF calculations.

7 $$\mathrm{absolute}\;\mathrm{error}\;\left(\%\right)=\left|F_{G}-F_{K}\right|$$

8 $$\mathrm{relative}\;\mathrm{error}\;\left(\%\right)=\frac{\left|F_{G}-F_{K}\right|}{F_{G}}\times 100$$

where *F*_*G*_ is *F* considering the NP geometry, and *F*_*K*_ is calculated using the Kanaras model.

#### 4.3.2 BCC NPs

For BCC structures, *F* is calculated and compared with the Kanaras model in Figure 3a. Due to their lower atomic packing density, BCC structures generally exhibit smaller *F* values than FCC or HCP structures, making them more consistent with the Kanaras model. However, trends in absolute and relative errors (Figure 3b,c) follow a similar pattern, with relative errors reaching 30–40% in larger NPs.

**Figure 3:**
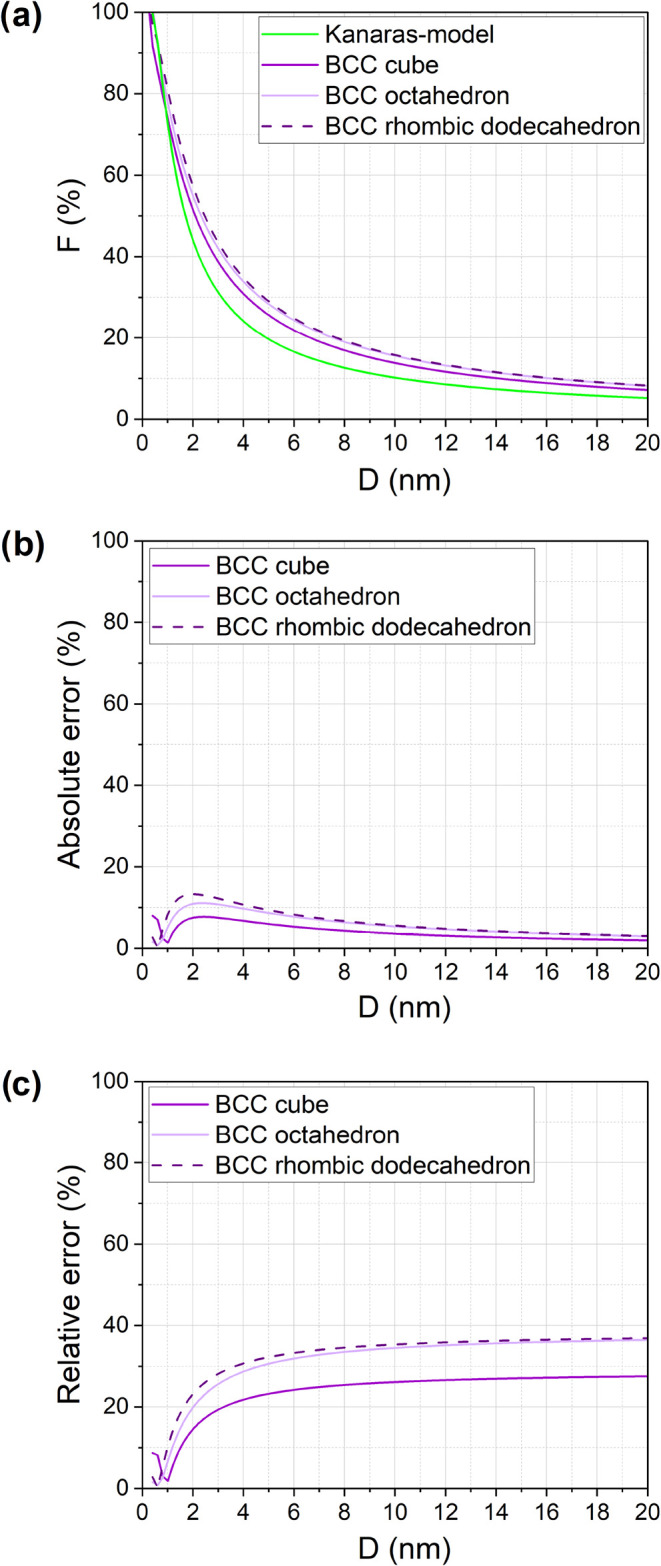
Comparison of (a) calculated fraction of surface atoms for BCC crystal structure with different shapes, (b) absolute error, and (c) the relative error in comparison to Kanara’s model (*r* = 0.124 nm).

Interestingly, the absolute error remains below 10% for all geometries as NP size increases, yet the relative error can be substantial. For 2 nm BCC NPs, regardless of the NP geometry, the maximum absolute error reaches 15%.

A fundamental requirement for consistency in surface fraction calculations is that both our model and the Kanaras model should converge to *F* = 100% for sizes below 1 nm. This is because, at sizes below 1 nm, NPs consist of small clusters of atoms (e.g., 55 Pd atoms for a 1 nm Pd cluster^24^) where nearly all atoms reside on the surface.

#### 4.3.3 HCP NPs

For two geometries of the HCP structure, the *F* is calculated and compared with the Kanaras model, as illustrated in Figure 4. The hexagonal bipyramid deviates significantly from a spherical shape, leading to an increased *F*, while the truncated hexagonal bipyramid retains higher sphericity and aligns more closely with the Kanaras model.

**Figure 4:**
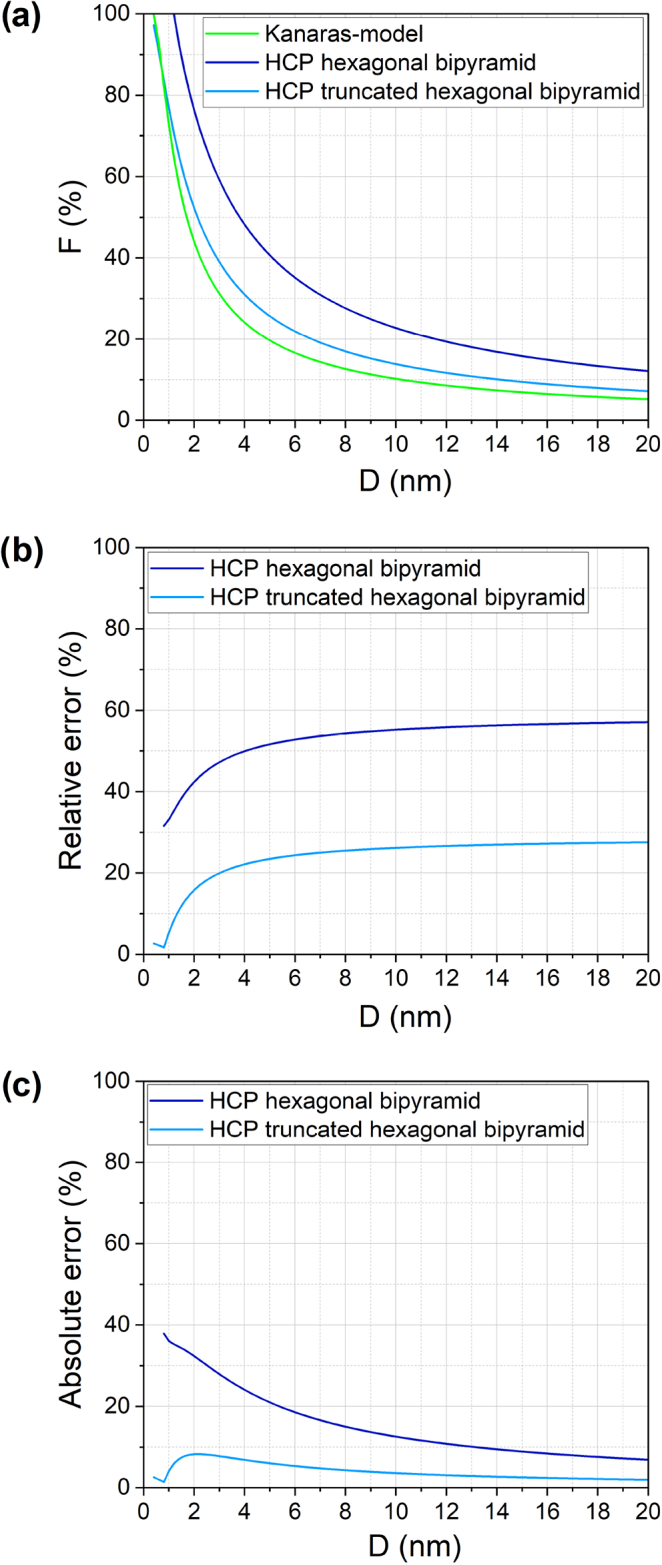
Comparison of (a) calculated fraction of surface atoms for HCP crystal structure with different shapes, (b) absolute error, and (c) the relative error in comparison to Kanara’s model (*r* = 0.124 nm).

Absolute error trends (Figure 4b) mirror those of FCC and BCC structures, where discrepancies decrease as NP size increases. However, relative errors (Figure 4c) remain high, exceeding 30% for truncated hexagonal bipyramids and reaching 60% for hexagonal bipyramids. Given that sphericity is lower for hexagonal bipyramids, a larger deviation in *F* is expected.

The results presented here provide a systematic understanding of how NP geometry influences *F*. Since TOF is directly proportional to the number of surface sites, any miscalculation in *F* will introduce errors in catalytic activity assessments. The relative errors observed in polyhedral NPs indicate that assuming spherical particles for TOF determination can significantly misrepresent catalytic efficiency.

In the next section, we apply this model to TOF calculations from CHC experiments, enabling a direct comparison of theoretical predictions with experimental observations.

### 4.4 Effect of Geometry on the TOF Calculation

Having established the influence of NP geometry on *F*, we now evaluate its impact on TOF calculations using the CHC reaction over the Ni-γAl_2_O_3_ catalyst as a model reaction. This allows for a direct comparison between TOF values derived from the spherical approximation and those incorporating the actual NP geometry.

The STEM-HAADF image of the Ni-γAl_2_O_3_ is illustrated in Figure 5a, highlighting the morphology of Ni NPs. Elemental analysis and the corresponding NP size distribution histogram are presented in Figure S1, indicating an average NP size of 9.9 ± 1.4 nm. As evident from Figure 5a and in agreement with previous studies,^32–35^ the FCC crystalline structure of Ni NPs adopts an octahedral geometry, a consequence of the thermodynamic stability of {111} facets.

**Figure 5:**
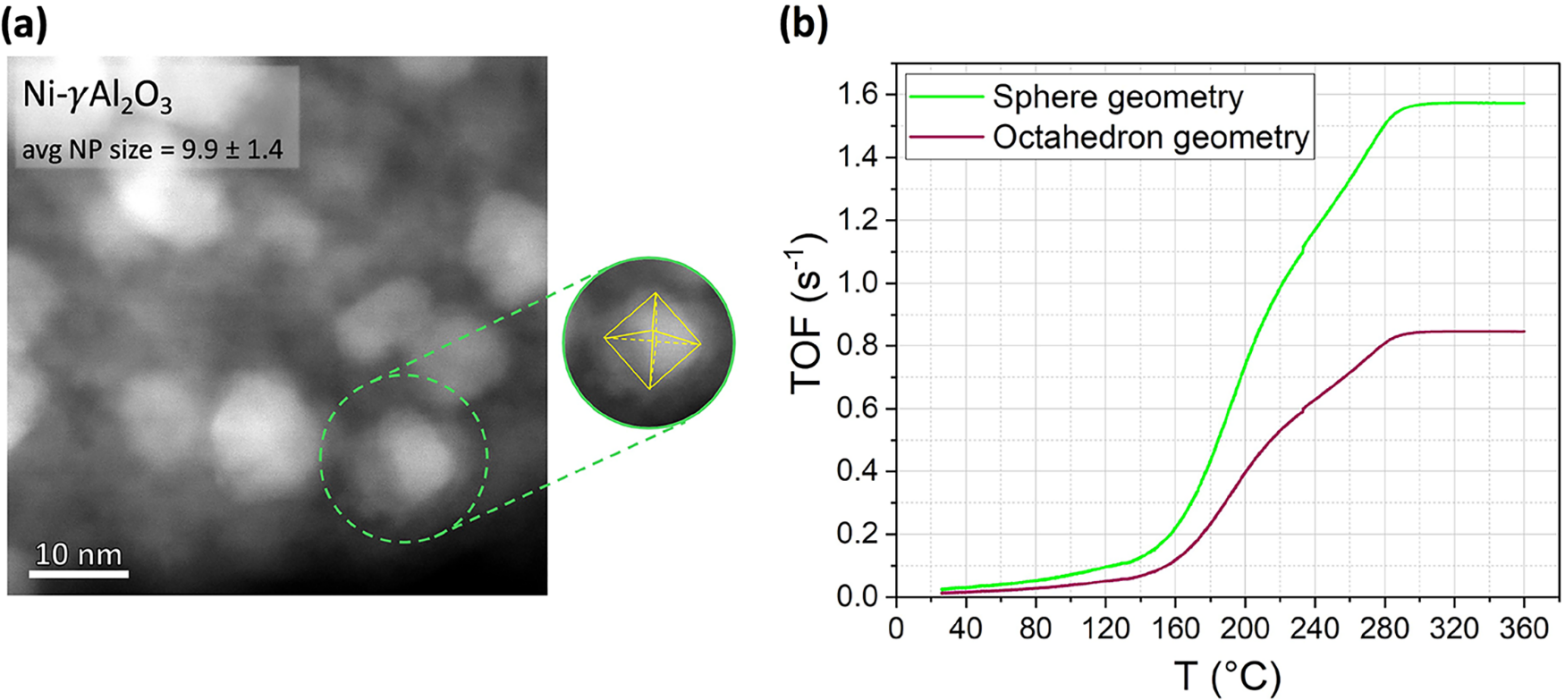
(a) STEM-HAADF image of Ni-γAl_2_O_3_. (b) TOF (S-1) of H2 during the CHC reaction over Ni-γAl_2_O_3_ catalyst as a function of temperature, considering the NPs geometry.

In situ X-ray diffraction (XRD) graph of Ni-γAl_2_O_3_ is provided in Figure S2. The results show the formation of pure metallic Ni after the reduction procedure under a H_2_ atmosphere. Using the Debye–Scherrer equation, the average crystallite size is calculated to be 11.8 nm. Comparing this value with the average particle size obtained by STEM confirms the formation of single-crystal Ni NPs. Therefore, during the CHC reaction, the individual metallic Ni NPs are exposed to the reactive gas.

Although the STEM-HAADF image in Figure 5a shows faceted Ni NPs, which are compatible with octahedral morphology, it should be noted that some degree of size and shape distribution or aggregation cannot be excluded. Such heterogeneity introduces uncertainty in dispersion estimation, a limitation inherent to all TOF calculation methods, including spherical, half-spherical, and geometry-specific models. The present analysis is based on the dominant observed morphology and aims to quantify the systematic error associated with assuming spherical geometry in such cases.

For the Ni NP with an average size of 9.9 nm, *F* is calculated using both the Kanaras model (based on spherical geometry) and octahedral geometry, yielding significantly different values of 10.3% and 19.2%, respectively.

When these values are used to calculate the TOF of H_2_, the results reveal that the spherical approximation overestimates TOF by 86% across the investigated temperature range (Figure 5b). This significant error primarily stems from the underestimation of *F* when assuming a spherical NP geometry. This suggests that accurate geometrical modeling is essential for catalytic performance predictions. Additionally, the observed deviations emphasize the importance of using geometry-specific models in kinetic studies, particularly when interpreting TOF data across different NP morphologies.

An underlying assumption in the current implementation is the use of static geometry based on prereaction STEM images. The potential evolution of NP geometry under reaction conditions is a known limitation that affects all approaches relying on dispersion-derived TOF calculations. The present model is not restricted to prereaction geometries and can be applied to any defined NP geometry and size, provided reliable structural parameters are available, whether obtained before, during, or after the reaction. In this context, the model can incorporate geometries obtained from in situ characterization^36^ or predictive modeling approaches.^37,38^

It should also be noted that, while TOF-based benchmarking is influenced by multiple experimental parameters, such as flow rate, catalyst-to-reactant ratio, reactor configuration, and support properties, these sources of variability are beyond the scope of the present work. The proposed model focuses on a specific and quantifiable contribution to TOF uncertainty: the effect of NP geometry on dispersion estimation. Geometry introduces a systematic bias that can be corrected when structural information is available. In this context, the model serves as a complementary tool to improve the internal consistency of TOF calculations, particularly for catalysts with well-defined morphologies.

Future work may focus on developing more refined models that account for varying NP geometries, anisotropic surface reactivity, and specific crystallographic facets. Such insights could pave the way for tailored catalyst design, optimizing catalytic performance by leveraging morphology-dependent reactivity.

## 5 Conclusion

In summary, we quantified the effect of geometry on the fraction of surface atoms (*F*) calculations. While we confirmed that the Kanaras model provides the most accurate estimation of *F* for spherical NPs, it fails for polyhedral structures, leading to a significant underestimation of surface site availability. To address this limitation, we developed a method to calculate *F* for NPs across multiple crystal structures (FCC, BCC and HCP) and various geometries, incorporating atomic-level details such as interatomic spacing. This approach enables precise evaluation and comparison of catalytic activity in heterogeneous catalysis by accounting for the shape of the catalyst NPs.

We applied the model to the catalytic H_2_ combustion (CHC) reaction over Ni-γAl_2_O_3_, and we demonstrated that assuming spherical geometry for Ni octahedrons overestimates the TOF of H_2_ by up to 86%. These results underscore the critical need for geometry-specific models to ensure accurate TOF calculations and reliable catalytic performance comparisons. The proposed method provides a robust framework for minimizing errors, enhancing the accuracy of kinetic studies, and facilitating the design of more efficient, morphology-optimized catalysts.

## Supplementary Material



## Notes

STEM HAADF-EDX images and Ni NP size distribution histogram are provided. Moreover, an in situ XRD analysis of Ni-γAl_2_O_3_ is provided, which shows the formation of metallic Ni after the reduction procedure. Using the Debye–Scherrer equation, the average crystallite size is calculated (PDF)
