# Supplementary material for: The Critical Role of Nanoparticle Geometry in Turnover Frequency Calculation
Source: ACS Meas Sci Au. 2025 Oct 14;5(6):1001–9. doi: 10.1021/acsmeasuresciau.5c00130 (PMC12715636; doi:10.1021/acsmeasuresciau.5c00130)
Supplement: Supplementary file 1 [file tg5c00130_si_001.pdf]

Supplementary Information

The Critical Role of Nanoparticle Geometry in Turnover Frequency Calculation

Zohreh Akbari[1,2], Loris Lombardo[1,2], Andreas Zuttel[1,2]

1 Laboratory of Materials for Renewable Energy (LMER), Institute of Chemical Sciences and Engineering (ISIC), Basic Science Faculty (SB), École Polytechnique Fedérale de Lausanne (EPFL) Valais/Wallis, Energypolis, Rue de l'Industrie 17, CH-1951 Sion, Switzerland

2 Empa Materials Science & Technology, CH-8600 Dubendorf, Switzerland

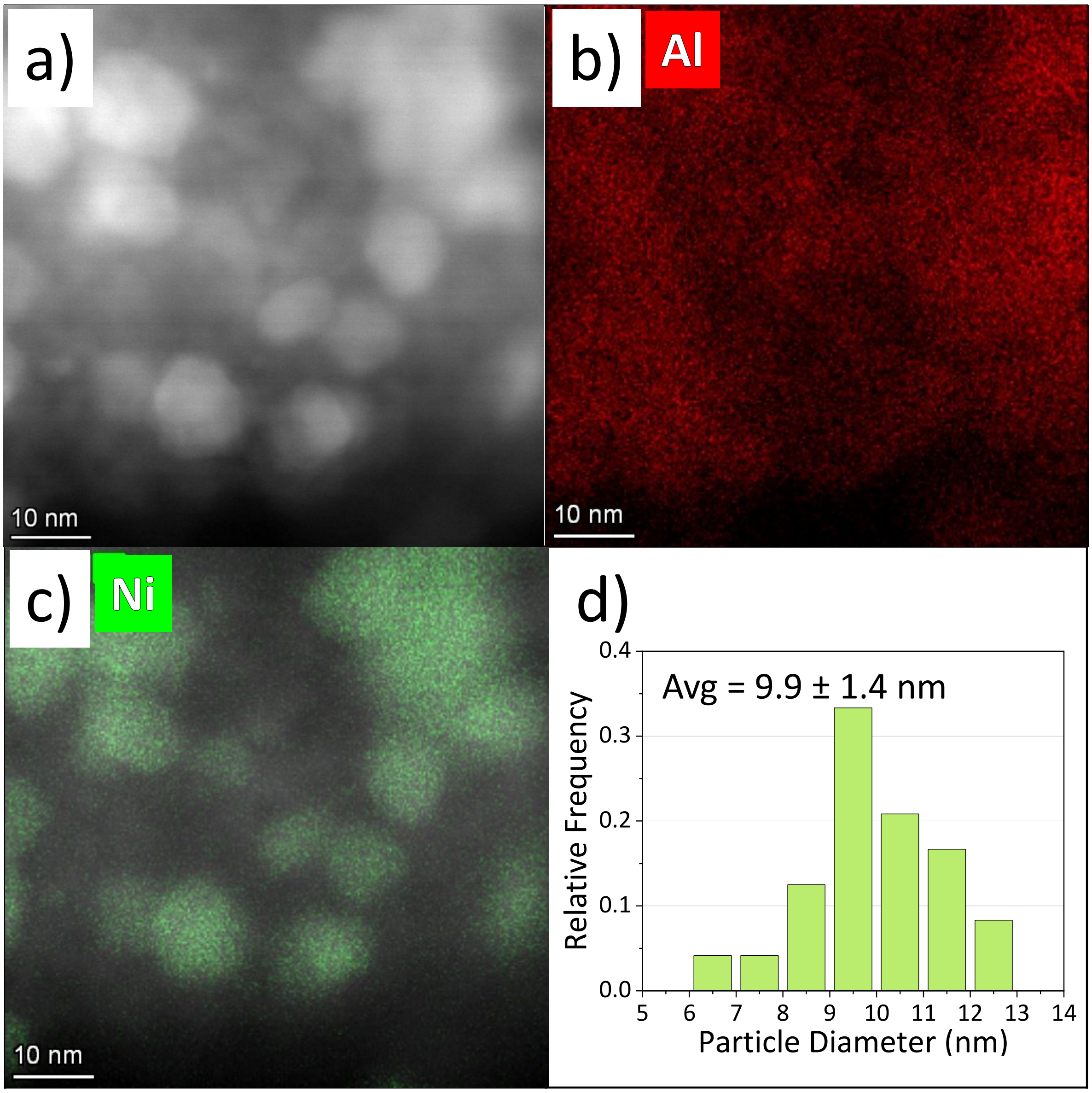

Figure S 1. a) HAADF-STEM image, b) Al, c) Ni elemental map, and d) size distribution of 10 wt% Ni supported on γAl_2O_3.

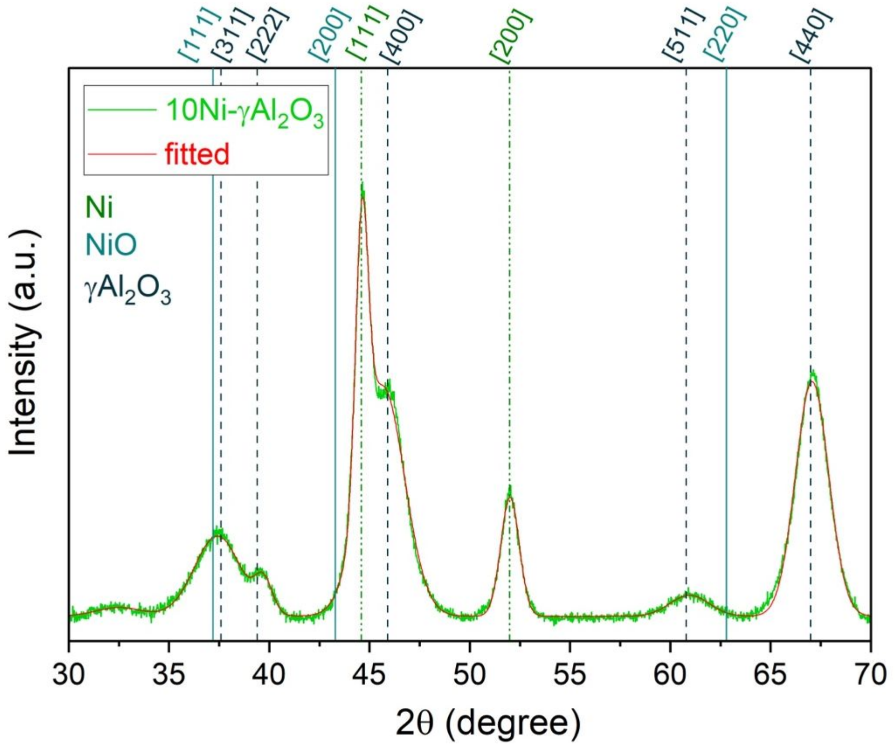

Figure S2. In-situ XRD graph of 10Ni-γAl_2O_3 at 400 °C under H_2.
